# Supplementary material for: Regionalization for health improvement: A systematic review
Source: PLoS One. 2020 Dec 22;15(12):e0244078. doi: 10.1371/journal.pone.0244078 (PMC7755212; doi:10.1371/journal.pone.0244078)
Supplement: S1 Appendix — (DOCX) [file pone.0244078.s001.docx]

**S1 Appendix - FULL-TEXT ARTICLES EXCLUDED LIST, WITH REASONS**

|  | **Articles excluded** |
| --- | --- |
| No available text | Doron and Ron, 1973; Lapre, 1975; Meise et al., 2000; Margolis et al., 2011; Junqueira and Motta, 2012 |
| Not from the health area | Okuda and Thomson, 2007; Pearce, Mawson and Ayres, 2008; Charron, Dijkstra and Lapuente, 2014; Ece, 2017 |
| Incompatible study design | Gosselin, 1984; Mawson and Spencer, 1997; Wyss and Lorenz, 2000; Jommi, Cantù and Anessi-Pessina, 2001; Keinert, 2001; Frankish et al., 2002; Hill and Wooldridge, 2002; D’Alva, 2004; Rose, 2004; Guimarães and Giovanella, 2004; Lima and Rivera, 2006; Wetta-Hall et al., 2007; Stoto, 2008; Spedo, da Silva Pinto and Tanaka, 2010; Wiktorowicz et al., 2010; Morrow et al., 2012; Rahb, Al-Sabri and Chitme, 2013; Santos, 2013; Vargas et al., 2015; Fleßa, 2015; Bleijenberg et al., 2016; Colombo, Garcia-Goni and Schwierz, 2016; Pereira and Tomasi, 2016; Vrangbaek, 2016; Duenas Fernandez, 2016; Frank and Marsden, 2016; Kim et al., 2017; Moreira, Ferré and Andrade, 2017; Nuti et al., 2017; Yoshiura et al., 2017; Kaselitz, Rana and Heisler, 2017; Nxumalo et al., 2018; Pereira, Lima and Machado, 2018; Ribeiro et al., 2018; Silva, 2018 |
| No clearly defined intervention | Luft, Bunker and Enthoven, 1979; van der Zwaan, 1987; Castells Oliveres, 1987; Cruz, 1992; Alisjahbana et al., 1995; Neves, 2001; Veenstra, 2002; Wainess et al., 2003; Cunha, 2005; Abdullah and Stoelwinder, 2007; Assis et al., 2009; Petersen et al., 2009; d’Avila Viana, de Lima and Ferreira, 2010; Guimarães, 2011; Luke and Muller, 2011; Perry et al., 2012; Schoos et al., 2014; Toth, 2014; Barreto Junior, 2015; Kildea et al., 2016; Kuriakose et al., 2016; Mancuso and Valdmanis, 2016; Nuti et al., 2016; Pereira et al., 2016; Macedo et al., 2017; Viana et al., 2017; Caimo, Pallotti and Lomi, 2017; Gallego et al., 2018; Paschoalotto et al., 2018; Perinazzo, 2018; Shukla, Khanna and Jadhav, 2018; Tovani-Palone, Formenton and Bertolini, 2018; Vicentine et al., 2018; Chang et al., 2018; Devereaux et al., 2020; Hansen et al., 2020. |
