# Supplementary material for: Regionalization for health improvement: A systematic review
Source: PLoS One. 2020 Dec 22;15(12):e0244078. doi: 10.1371/journal.pone.0244078 (PMC7755212; doi:10.1371/journal.pone.0244078)
Supplement: Attachment — Submitted filename: response_to_reviewers.docx [file pone.0244078.s004.docx]

Dear Editor,

We are thankful for the contributions to our manuscript “Regionalization for health improvement: a systematic review”. This evaluation is of utmost importance to help us improve and enrich our paper. We present the responses addressing each of the questions below.

Best regards,

The authors

**Comment 1, Editor:** “We noticed you have some minor occurrence of overlapping text with the following previous publication(s), which needs to be addressed: Ke, K. M., W. Hollingworth, and A. R. Ness. "The costs of centralisation: a systematic review of the economic impact of the centralisation of cancer services." European journal of cancer care 21.2 (2012): 158-168. The text that needs to be addressed involves a paragraph of the Discussion. In your revision ensure you cite all your sources (including your own works), and quote or rephrase any duplicated text outside the methods section. Further consideration is dependent on these concerns being addressed”.

**Our response:** We are thankful for the time spent on correcting our manuscript and the contributions mentioned. The paragraph in question was rewritten and properly cited.

**Comment 2, Editor: “**Please provide an amended Funding Statement that declares *all* the funding or sources of support received during this specific study (whether external or internal to your organization) as detailed online in our guide for authors at http://journals.plos.org/plosone/s/submit-now”.

**Our response:** The authors received no specific funding for this work.

**Comment 3, Editor:** “Please state what role the funders took in the study. If any authors received a salary from any of your funders, please state which authors and which funder. If the funders had no role, please state: "The funders had no role in study design, data collection and analysis, decision to publish, or preparation of the manuscript."

**Our response:** The authors received no specific funding for this work.

**Comment 4, Editor:** “Please complete your Competing Interests on the online submission form to state any Competing Interests. If you have no competing interests, please state "The authors have declared that no competing interests exist.", as detailed online in our guide for authors at http://journals.plos.org/plosone/s/submit-now

This information should be included in your cover letter; we will change the online submission form on your behalf.”

**Our response:** We included the sentence "The authors have declared no conflict of interest.", as requested.

**Comment 5, Editor:** “Please include a copy of Table 33 which you refer to in your text on page 16”.

**Our response:** We believe there was a typing mistake, as the correct Table should be number 3. Table 3 is located in page 13.

**Comment 6, Editor:** “Please include captions for your Supporting Information files at the end of your manuscript, and update any in-text citations to match accordingly. Please see our Supporting Information guidelines for more information: <http://journals.plos.org/plosone/s/supporting-information>.”

**Our response:** The captions were included and the citations were updated.

**Comment 1, reviewer 1:** “The article entitled, “Regionalization for health improvement: a systematic review” presents an ambitious research question, aiming to summarize interventions that have been successful at the organization of health care services in order to guide future recommendations. To answer this question, the authors have chosen a literature review to find original research describing the impact of such reorganization policies. The results summarize the articles found in this search and conclusions remain broad as the study results are quite varied. There are a few overall themes which need to be better addressed by the authors in order to meet the aims they have set out in this study.

1. The definition of regionalization used in this research does not appear to match more common definitions. Traditionally, regionalization refers to the centralization of health services in one location – such as high volume or specialized centers. The authors use of the term seems to be more in the vein of organization of health services. This warrants renaming the study and substituting the term regionalization for “optimal organization” or some alternative phrasing.”

**Our response:** We are thankful for the time spent on correcting our manuscript and the contributions mentioned. We have understood the argument of the reviewer and consider it interesting, however the subject of regionalization is still highly debated, and as such there is little consensus on the subject as a public policy. Certain public health systems have decided to regionalize their services. Canada and Brazil are prominent examples of countries that adopted the policy of regionalization in health as a means to organize healthcare services (Marchildon, 2019). Whenever the literature indicates that one of the characteristics of regionalization is decentralization, it is referring to “to decentralize responsibility for the administration of major health programs and to centralize authority over the operation of these same programs by eliminating hospital boards and other local health agencies” (Baker et al, 2017). According to Reamy (1995), decentralization is an important subject to regionalization. It implies in the transference of any degree of authority and responsibility towards a lower level or region. In some cases, decentralization and regionalization occur simultaneously, as the region might be decentralized in terms of governance but centralized in terms of providing hospital services, as mentioned by the reviewer. It should also be noted that when characterizing regionalization as decentralized we are referring to reorganization at all healthcare levels (primary, secondary and tertiary care), not just a specific kind or level of care such as hospital care. Moreover, Brazil, Canada and Italy, for instance, have adopted the policy of regionalizing their systems of healthcare services together with the reorganization of healthcare networks – also known in other countries as integrated service networks, regional networks, integrated services, and integrated care (Viana et al, 2018). Healthcare networks provide a new logic for the organization of health services, defined as a set of actions and services articulated at increasing levels of complexity in order to ensure the integrality of healthcare.

References:

1. Marchildon G. The integration challenge in Canadian regionalization. Cadernos de Saúde Pública. 2019; 35 Sup 2:e00084418.
2. Barker, P., & Church, J. (2017). Revisiting Health Regionalization in Canada: More Bark Than Bite? International Journal of Health Services, 47(2), 333–351. <https://doi.org/10.1177/0020731416681229>).
3. Reamy J. Health Service Regionalization in New Brunswick, Canada: A Bold Move. International Journal of Health Services. 1995; 25(2): 271–282.
4. Viana Ana Luiza d’Ávila, Bousquat Aylene, Melo Guilherme Arantes, Negri Filho Armando De, Medina Maria Guadalupe. Regionalização e Redes de Saúde. Ciênc. saúde coletiva [Internet]. 2018 June; 23( 6 ): 1791-1798.

**Comment 2, reviewer 1:** “2. The methodology needs to be revised as detailed in comments below. 3. The organization of the results with use of the terms regionalization, rationalization, and decentralization need to be presented differently.”

**Our response:** As mentioned in the previous item, the results were presented according to the concept utilized by authors studying regionalization, which in turn is the integrated organization of a healthcare system with several coordinated functions that serves a specific territory wherein a regional governance structure is responsible for providing and administrating health services of that specific region (Aerde, 2016). For this reason, we present the results in the dimensions: decentralization, rationalization, coordination, and governance.

Reference:

5. Aerde, JV. Has regionalization of the Canadian health system contributed to better health?. Canadian Journal of Physician Leadership, 2016; 2(3):65-70.

**Comment 3, reviewer 1: “4.** **I would recommend focusing on either health outcomes or distribution of resources instead of the effects of health system reorganization on both”.**

**Our response:** We have understood this recommendation and are thankful for the suggestion, however, the choice for presenting the results of regionalization in the allocation of resources and in its efficacy of health outcomes was made due to the number of studies identified for both. Several studies demonstrate the importance of centralizing high complexity services and how this centralization is necessary to optimize resources for health; however, few are those that describe the results of adopting regionalization as a service organization policy. As such, we opted to keeping both results to present the evidence identified in the literature concerning the adoption of the regionalization policy in health in order to report the decision-making to administrators that might be interested in adopting a similar policy in their countries.

**Comment 4, reviewer 1:** “Abstract: Page 2 – The first sentence of the abstract is unclear to me and does not appear to match the aims of the study. “The health services regionalization” may be better worded differently. Perhaps the intent is to describe interventions aimed at the regionalization of health services and describe their impact. The second sentence is somewhat repetitive of the first and similarly should be reworded. The health and management indicators mentioned should also be more clearly defined further in the study. Further in the abstract it states “the health regionalization system” which is also an unclear term and should be better defined. I would recommend defining regionalization in the abstract. “The results of the studies included as a highly recommended intervention showed that regionalization was effective within the decentralization and rationalization of resources” – this sentence should be made more clear and again the terms regionalization, decentralization, and rationalization should be more clearly defined and do not appear to be used effectively here. “The most investigated intervention was the adoption of the strategy of concentration of procedures in high-volume hospitals,” this is more commonly the definition of regionalization”.

**Our response:** We included the excerpt “Regionalization is the integrated organization of a healthcare system, wherein regional structures are responsible for providing and administrating health services in a specific region. This method was adopted by several countries to improve the quality of provided care and to properly utilize available resources” in the abstract. As for the use of the term decentralization, we have previously justified the reason for our choice.

**Comment 5, reviewer 1:** “Introduction: Page 2 – please re-evaluate the definition of regionalization used in the paper. Page 3 – The first paragraph starts by defining decentralization as part of regionalization, whereas more commonly they are considered to be opposing ways of organization health services. Page 3 – again would not consider decentralization as part of regionalization. This should instead be changed to describe the organization or distribution of health services.”

**Our response:** The definition of regionalization was included as understood by the authors. In order to address the reviewer’s request and clarify the utilized concept, we included the following excerpt:

“The literature shows the Dawson Report as one of the first and main documents to concern regionalization in 1920. The author pictured an integrated healthcare system wherein the prevention of diseases and healthcare promotion would be coordinated, including primary, secondary and tertiary care of both short and long term serving to organize health services in territories specifically selected for such services^[3]^. England, Canada and Brazil are amidst the countries that used Dawson’s ideals to develop health services regionalization policies to integrate the coordination of institutional and community integration in order for the community to participate, and to transfer the authority of management concerning healthcare programs to regional level. The centralization of governance by eliminating local healthcare entities and agencies in these countries was followed by the decentralization of responsibility towards providing most health services^[4]^. Likewise, there is no consensus on the concept of regionalization. In federative countries, the concept that is closest to regionalization is: the integrated organization of a healthcare system with several coordinated functions that serves a specific territory wherein a regional structure would be responsible to provide and administrate healthcare services^[5,6]^.

According to Marchildon^[7]^, the goal of regionalization is to integrate and rationalize healthcare services, to promote evidence-based practice, to decentralize resources and decision-making, and to switch focus and resources towards the prevention of ilnesses and promotion of health”.

We also reworded the last paragraph:

“This study thus sought to identify and characterize effective interventions to improve health and management indicators within the scope of the regionalization of health services. A systematic review was conducted to answer the following question: what are effective interventions to improve health and management indicators within the health services regionalization?”

**Comment 6, reviewer 1:** “Page 3, last paragraph – this is not complete and again goes back to the comment on the abstract on how this is not a clear statement. “The aim of this study is to identify and characterize effective interventions to improve health and management indicators within the health services regionalization. A systematic review was conducted to answer the following question:”

**Our response:** Indeed, the last paragraph was incomplete. We have altered it to:

“This study thus sought to identify and characterize effective interventions to improve health and management indicators within the scope of the regionalization of health services. A systematic review was conducted to answer the following question: what are effective interventions to improve health and management indicators within the health services regionalization?”.

**Comment 7, reviewer 1:** “Methodology: Page 4 – please redefine the question”.

**Our response:** As previously justified, we used the terms regionalization and decentralization in a joint manner and not as opposite concepts, as stressed by the reviewer, due to the concept of regionalization adopted in the public policies of countries such as Canada, Brazil and Italy. We agree that centralization of highly specialized care is a common and important strategy in several countries, however we are referring to political and administrative decentralization of the healthcare system, thus justifying the question and search utilized.

**Comment 8:** “Page 4 – Outcomes – the health management outcomes need some basis for where these came from. Ambulatory care-sensitive conditions should be defined.”

**Our response:** The following excerpt was included:

“The Taxonomy of Decisions of McMaster University was used to define effects on management. This taxonomy allows for better standardization of health descriptors, as it proposes useful patterns for ideal decision-making and provides tools to take good practical decisions^[15]^”.

**Comment 9, reviewer 1:** “Page 4 – Search strategy – the search string should instead include OR between these brackets instead of AND to cast a wider net, at least between the two first strings of searches. Especially considering the regionalization and decentralization terms used. There are also additional synonymous terms which should be used which have been omitted – including devolution which is used in place of decentralization and centralization which is synonymous with regionalization. Here is an example of a more broad search for regionalization and decentralization and I would recommend all these terms be used at a minimum. Decentralization/Regionalization Search String (<https://www.ncbi.nlm.nih.gov/pubmed/31657175>) (district hospital) OR (community health center) OR (primary health centre) OR (primary health center) OR (community facility) OR rural OR decentralization OR decentralized OR decentralized OR regionalization OR regionalized OR regionalised OR (First-level hospital) OR (referral hospital) OR (specialised hospital) OR (specialized hospital) OR (devolution) OR (devolved) OR (devolve) OR (devolvement) OR (devolving) OR (centralization) OR (centralized) OR (centralised)”

**Our response:** As previously justified, “regionalization AND decentralization” was used due to the concepts of regionalization and decentralization utilized.

**Comment 10, reviewer 1:** “The last search string on outcomes should include mortality and health outcomes more broadly”.

**Our response:** The Taxonomy of decisions of the McMaster University was used for choosing the terms utilized in the search, as previously explained.

**Comment 11, reviewer 1:** “Page 8 – please define the GRADE system in 1 or 2 sentences and it’s application to this study more clearly.”

**Our response:** The paragraph concerning GRADE was altered to:

“The Grading of Recommendations Assessment, Development and Evaluation (GRADE) was adopted to evaluate the results. The GRADE system is a transparent structure for developing and presenting evidence summaries, providing a systematic approach for recommendations to improve clinical practice, with clear and concise information over the quality of evidence (the degree to which a given result might be trusted) and the reliability of the recommendation^[16]^. With GRADE, the interventions identified during systematic review were stratified as “Level of recommendation: very low recommendation; low recommendation; moderate recommendation, and; high recommendation”.

**Comment 12, reviewer 1:** “Results. Page 9 – The first two paragraphs are repetitive of the first table and can be shortened.”

**Our response:** The first two paragraphs were revised as suggested by the reviewer.

**Comment 13, reviewer 1:** “Page 9 – The paragraph on the GRADE system is descriptive of methods and should be included there”.

**Our response:** Information concerning GRADE was included in the methodology, as previously reported.

**Comment 14, reviewer 1:** “Table 3 – the dimension and interventions chosen here need more of a basis. Either in the introduction or methods – there should be a better description of how these dimensions were settled on”.

**Our response:** The dimensions were defined in the introduction, as follows:

“Studies show that regionalization needs organizational arrangements to achieve its proper functioning^[9,10]^, namely: i) coordination: concerns the integration, sharing, articulation and decision-making based on norms, legal or not, whose actors agree to share decisions and tasks; in forums and political mechanisms for intergovernmental negotiation; the functioning of representative institutions; in the coordinating and/or inducing role of Brazilian government^[11]^; ii) decentralization: defined as the delegation or devolution of certain responsibilities and functions, broadening the view on the autonomy of other federative entities^[12]^; iii) rationalization: it is defined as a resource utilization tool aiming to increase the efficiency of services without bias towards the effectiveness of the actions^[13]^; iv) governance: it is defined as the actions and means adopted by society to organize itself to protect and promote the health of the population, seeking to harmonize the decisions and actions of different actors in favor of the equity and sustainability of health systems^[14]^”.

**Comment 15, reviewer 1:** “Define the “polo hospital” in table 3”

**Our response:** We have changed the term “polo hospital” to “high-volume hospital”.

**Comment 16, reviewer 1:** “The rationalization term used here is synonymous with regionalization broadly in the literature”.

**Our response:** Regionalization in health is understood as a policy to organize services in an integrated and coordinate manner, including political-administrative transference to specific territories and optimally allocating resources to meet health needs. As such, rationalization is an important concept under the scope of health regionalization, however, regionalization itself is broader than the effective utilization of health resources.

**Comment 17, reviewer 1:** “Discussion. Page 19 - “The results of the studies included as a highly recommended intervention showed that regionalization was effective within the decentralization and rationalization of resources.” Can this be differently worded? Unclear of authors’ intention with this statement.”

**Our response:** As previously justified, we presented the results in the dimensions decentralization, rationalization, coordination, and governance as they are key concepts for health regionalization.

**Comment 18, reviewer 1:** “Because the results of the articles are so varied, there is really only one reasonable conclusion that can be drawn, as the authors point out – the concentration of procedures in high volume hospitals has shown favorable health outcomes. This has been shown in previous studies, as the authors point out, so it is unclear what new perspective this study brings to the literature.”

**Our response:** Despite other results not including clear evidence, it is important to document and discuss them as possible results of the implementation of a regionalization policy in the country. It must be noted that in the field of public policies uncertainty or lack of evidence is not comparable to their inefficacy. Unfortunately, we still find it difficult to document public policy cases implemented in certain countries to inform the local experience of other countries. However, evidencing actions with potential effect on public health contributes to the reduction of the know-do gap and helps the decision-making process in health.

**Comment 19, reviewer 1:** “Other limitations of the study include the literature is largely from the USA and HICs. There are only two low income countries included. The authors mention this in the last two paragraphs. Recommendations or insights are therefore only attributable to HICs. I would appreciate further discussion on the disparities in different health settings and why or why not such interventions recommended in this study may be successful in these areas.”

**Our response:** The following excerpt was included:

“Implications for politics

The first point to consider is that, although literature points to the possible effectiveness of regionalization, it must be considered that each country has its political, economic, and geographical structure, which makes it daring to propose a standard recipe for the implementation of health regionalization^[73]^. It's required that decision-makers, organized civil and academic society to evaluate the necessary changes and feasibility for the implementation of an evidence-informed policy, taking in consideration the uncertainties of the evidences and balancing different results^[74]^.”

**Comment 1, reviewer 2:** “The manuscript presents originality in its proposal and important relevance for health services regionalization. The authors were able to organize a methodological strategy that allowed them to argue that the difficulties of articulating health services regionalization in differents aspects resulted from dimmensions. Based on these results, they were able to elaborate a set of recommendations that can mitigate the articulation difficulties identified. I suggest the authors make clear the regionalization defines they are using in the manuscript. I recommend indicating the countries are using health regionalization and to indicate their success.”

**Our response:** We are thankful for the time spent on correcting our manuscript and the contributions mentioned. We have included the definition of regionalization used in the study, as you can see in **Comment 5, reviewer 1**.

**Comment 2, reviewer 2:** “Review if is correct in the end of introduction, it is looks like are missing some questions. I recommend use the standard Plos one Reviewer Guidelines.”

**Our response:** Following the reviewer’s observation, we have changed the last paragraph to:

“This study thus sought to identify and characterize effective interventions to improve health and management indicators within the scope of the regionalization of health services. A systematic review was conducted to answer the following question: what are effective interventions to improve health and management indicators within the health services regionalization?

**Comment 3, reviewer 2:** “The discussion of results follows the logic of the entire study, that is, an investigation about different realitys, certainly with few similarities in other latitudes. However, this does not prevent to identify aspects overlapping with the reality of other health systems.”

**Our response:** The following excerpt was included:

“Implications for politics

The first point to consider is that, although literature points to the possible effectiveness of regionalization, it must be considered that each country has its political, economic, and geographical structure, which makes it daring to propose a standard recipe for the implementation of health regionalization^[73]^. It's required that decision-makers, organized civil and academic society to evaluate the necessary changes and feasibility for the implementation of an evidence-informed policy, taking in consideration the uncertainties of the evidences and balancing different results^[74]^.”

**Comment 4, reviewer 2:** “I recommend a major revision of the English grammar, as this would make the text more comprehensible to all readers.”

**Our response:** We have contracted a certified language services provider to revise this paper and make it easier to understand for all readers.
