# Supplementary material for: Regionalization for health improvement: A systematic review
Source: PLoS One. 2020 Dec 22;15(12):e0244078. doi: 10.1371/journal.pone.0244078 (PMC7755212; doi:10.1371/journal.pone.0244078)
Supplement: Attachment — Submitted filename: response to reviewers.docx [file pone.0244078.s005.docx]

Dear Editor,

We thank you for the opportunity to have our manuscript **PONE-D-20-14125R1**, entitled **"Regionalization for health improvement: a systematic review"**, evaluated for publication by Plos One. We also would like to thank the reviewer for his/her consideration and time spent reviewing our manuscript.

Yours sincerely,

The Authors

**Reviewer 1, comment 1:** “The authors have made substantial improvements to the initial manuscript and I sincerely appreciate their consideration and response to my prior comments. However, the study still has some significant methodologic flaws.

While I appreciate the authors comments clarifying the concepts of regionalization and decentralization being used in this study, their search strategy for studies does not match the main objectives. One of my prior comments was to expand their search strategy as to not miss out on publications that may mention regionalization, the main focus of this current study, in isolation of the other terms that are mentioned in the search strategy: decentralization, rationalization. I would still strongly recommend revising the search strategy to more broadly search for regionalization in accordance to my prior comment. One way to limit the results further would be to use the regionalization terms then include a longer AND statement to get to the components of health structuring they are hoping to capture further: for instance regionalization (including all relevant terms) AND (decentralization terms OR coordination terms OR rationalization terms OR governance OR outcome terms). The current search strategy is severely limiting to papers that include regionalization AND decentralization AND organizational strategy/outcomes. It also appears several of the search terms would need to be expanded to include all relevant permutations. For example, "Mortality rate" OR "child mortality rate" are included, but not mortality and not appropriate MeSH terms for these.”

**Our response:** Thank you for taking the time to review our manuscript and for the helpful comments you have provided.

As it is a complex issue, we had previously included outcomes in the search strategy following the guide to a systematic review of the University of York, Chapter 3 - Systematic reviews of public health interventions. Understanding the reviewer's questioning, we redid the search strategy accordingly, excluding the outcomes. This change caused an increase in the articles identified from 657 to 3918. With the new search suggested, the strategy was used:

#1	MeSH descriptor: [Regional Health Planning]

#2	(regional governments):ti,ab,kw

#3	(regional healthcare):ti,ab,kw

#4	(areawide planning):ti,ab,kw

#5	(regional healthcare network):ti,ab,kw

#6	(comprehensive health planning):ti,ab,kw

#7	(annual implementation plans):ti,ab,kw

#8	(regional governments):ti,ab,kw

#9	(regionalization):ti,ab,kw

#10	OR{#1-#9} or #2 or #3 or #4 or #5 or #6 or #7 or #8 or #9

#11	(decentralization):ti,ab,kw

#12	(coordination):ti,ab,kw

#13	(co ordination):ti,ab,kw

#14	(rationalization):ti,ab,kw

#15	(governance):ti,ab,kw

#16	#11 or #12 or #13 or #14 or #15

#17	#10 and #16

Except for 3 studies, the studies previously included were identified in a new search. The selection by title and abstract was carried out in a paired and independent manner by four researchers, to comply with the established deadline. In total, 119 studies were selected for a full reading. The inclusion stage was also carried out in a paired and independent manner by two researchers, with 39 meetings the inclusion criteria established, that is, 6 more than the previous search. Because of the change in the search, we inform that the METHODOLOGY section of the article has also undergone changes, which are highlighted below:

**METHODOLOGY**

The following search strategy was used: ("regional health planning" OR "regional governments" OR "regional healthcare" OR "area wide planning" OR "regional healthcare network" OR "comprehensive health planning" OR "annual implementation plans" OR "regional governments" OR "regionalization") AND ("decentralization" OR "coordination" OR "co ordination" OR "rationalization" OR "rationaliz$" OR "governance).

There was no language or date restriction, and the search was conducted between February and March 2017 and updated in October 2020.

The inclusion criteria were studies that answered the PICOT question. Studies concerning systematic reviews methodology, overviews of systematic reviews, theoretical, qualitative, editorial studies and letters to the editor were excluded, as well as studies that did not address interventions related to clearly-defined organizational arrangements of regionalization, or that did not provide the full text of the study.

The systematic review followed the PRISMA for systematic review protocols. The protocol was previously registered on the Prospero platform (CRD42016042314).

The selection of studies was performed in two steps: i) selection by title and abstract by four independent reviewers (MR, ENS, JOMB and HES), and the differences resolved by consensus; ii) selection after reading of the studies in full by two independent reviewers (MR and ENS), with disagreements resolved by consensus.

We also made changes to the results, to describe the 6 studies included with the new search strategy:

**RESULTS**

The search found 3921 documents, of which 604 were duplicates. After reading the titles and abstracts, 486 documents were excluded. The reason for excluding this step is described in the Supplementary Material. After complete reading of the documents, 39 studies were selected to compose the systematic review. Figure 1 describes this process.

[…]

Table 1 summarizes the main characteristics of the studies included in this systematic review. Regarding the year of publication, there is a study published in the 1980s^[42]^, six in the 1990s^[30,31,33–35,38]^, seventeen in the 2000s^[17–20,27–29,32,37,41,43,46,47,52,54–56]^, fourteen in the 2010s^[22–24,26,36,39,40,44,45,48–51,53]^ and one in the 2020s^[21]^. As for the period studied, there is a wide variety. Studies^[17,29,32,37,41,42,46,50,51]^ with up to one year of analysis corresponded to 23.07% (n=9) of publications, followed by those with more than 10 years^[20,24,31,55]^ (10.25%; n=4) and those with one year of analysis^[27,33,38,56]^ (10.25%; n=4). Regarding the country of origin, 56.41% (n = 22) are from the United States^[18,20,22,23,27,29–31,35–39,41–43,45–47,52,55,56]^. Publications from Italy^[21,26,48]^ total 7.69% (n=3), while publications from Australia^[32,51]^, Brazil^[17,40]^, the Netherlands^[50,54]^ and Canada^[28,34]^ account for 5.12% (n=2) each. Zambia^[19]^, Uganda^[49]^ and a partnership between Canada and the United States^[33]^, and between Italy and Spain^[24]^, each of the publications with 2.56%. (n=1).

Regarding the study method, 11 distinct methods were identified, and the most frequent was the regression model^[17,20–22,24,27,29–31,33,35–38,42,43,46,47,52–56]^, in general, with 58.97% (n=23). However, it is noteworthy that the regression models varied between simultaneous, Poisson, linear, generalized linear equations with Poisson error structure, logistical, logistical and linear, hierarchical, multiple and Cox multivariate logistics. Ecological^[32,37,48]^ and cross-sectional studies^[40,49,51]^ add up to 7.69% each (n=3) and quasi-experimental studies^[44,45]^ accounted for 5.12% (n=2). The methods of cost-utility analysis^[18]^, proportional risk survival analysis^[28]^, cost-effectiveness analysis^[23]^, cost analysis^[50]^, Propensity score^[41]^, multiple comparison test^[34]^, cohort^[39]^ and Data Envelopment Analysis^[26]^ totaled 2.56% of the studies, each.

For quality assessment, the instrument of The Joanna Briggs Institute was used^[57]^. After weighting by two independent researchers, 58.97% of the studies obtained maximum quality assessment (8/8), while 23.07% obtained intermediate evaluation (7/8 or 6/8). Another 10.25% of the studies had regular evaluation (4/8) and 5.12% poor evaluation, with 0/8 points established by the instrument. The result of the quality assessment can be seen in detail in Table 2.

Also, minor changes were made, identified in the Marked-up copy. Four strategies were identified in the new studies included. Two studies addressed the rationalization dimension, with the strategy “Concentration of procedures in hospitals with high volume of production”.

Regarding the coordination dimension, the “Coordinated care organization” strategy was included based on the results of the new studies:

Within the “coordination” dimension were the interventions of “Integration of surgery and cancer therapy in the same physical facility”, “Provision of sanitary transport services between areas further from the high-volume hospital”, “Coordinated care organization”, “Outreach services in remote areas” and “Outpatient and psychosocial network integration”.

[…]

The third intervention refers to coordinated care organization, which are patient-centered integrated health services that are geographically defined with a reference site for population care. They are services of low and medium complexity that act as a gateway to outpatient care. Two different studies have been identified for depicting this type of intervention^[44,45]^.

The first one, carried out in France, performed a quasi-experimental study devised into two clusters: ‘everyday care’ and ‘coordinated care’ in the treatment of cancer patients who were on oral medication. The authors identified that the overall six-months survival rate was of 76% in the first cluster and of 87% in the latter (p = 0.064). No significant statistical changes were observed in the rates of disease progression, quality of life or treatment compliance^[44]^.

The second study was conducted in the United States of America (USA). It analyzed the trends in early prenatal care initiation and trends in prenatal care adequacy after the implementation of the coordinated care organization model. The authors found that the rate of early prenatal care initiation increased significantly, from 73.1% to 77.3%, while prenatal care adequacy from 65.9% to 70.5%. The effect of implementing CCO in prenatal care adequacy, however, was not significant. After a sensitivity analysis, the estimates remained constant^[45]^.

Regarding the decentralization dimension, a new study was included in the “Decentralization of care” strategy, being:

Six interventions were categorized as belonging to the “decentralization” dimension. The first, “Decentralization of care”, states that health care actions should be performed at the regional level, including four studies in this intervention.

[…]

The last study included in this intervention compared the health outcomes of patients who were tended by either traditional or decentralized teams. The analyzed supply variables showed that: patients who had been tended by decentralized teams experienced only half of the expected amount of treatment and of the annual outpatient doctor-patient interactions; but the amount of hospitalizations was 17.3% higher among patients who had been given decentralized care. The annual costs of hospitalizations ranged from 886 to 3223 Euros for decentralized teams and 2038 Euros for central outpatient care. The costs of psychotropic and outpatient treatment, however, are significantly higher for the central outpatient department (1,050 Euros per year for the former and 781 Euros for the latter). The authors conclude that the number of persons admitted has slightly increased, as did the number of admissions. Despite that, the number of admissions per person admitted to the hospital did not suffer any alterations^[53]^.

Still in this dimension, the new strategy “Fiscal decentralization” was identified, including the excerpt:

The fifth intervention was called "fiscal decentralization" and refers to the fiscal autonomy of regional health authorities (RHA). It is a financing mechanism, a quasi-market model with purchaser-provider. This model allows RHA to be responsible for delivering a basic package of health care services through a network of population-based local health authorities as well as public and private accredited hospital providers. A study conducted a convergence analysis to assess whether fiscal decentralization could improve two selected health indicators: the infant mortality rate (IMR) and the increase in life expectancy at birth (LEB). The authors claim that there is no clear indication of σ convergence or long-term σ divergence for both outcomes in patients’ health. According to the authors, the effects of fiscal decentralization do not seem to demonstrate a systematic dynamic.

For both outcomes in patients’ health, all axis exhibits a negative slope (always statistically significant at 1% level), which is consistent with the hypothesis of β-convergence. Furthermore, as the level of decentralization increases, the slope of the axis (β coefficient) increases. This indicates the beneficial effect of higher degrees of fiscal decentralization in improving the convergence process. However, the fact that the axis intersect in the analysis indicates that the decentralization growth ratio is sensitive (moderate) to the level of health outcomes of the geographical region being analyzed. As the IMR decreases and LEB increases, the fiscal decentralization’s efficiency in contributing to the reducing of the IMR or the increase of LEB is reduced^[21]^.

In the DISCUSSION, two new paragraphs were included, which are transcribed below:

Despite the positive results of regionalization, Cavalieri & Ferrante (2020) warn that they have not identified any obvious connections between fiscal decentralization and improvements in a population’s health. This result is in agreement with the published literature on the subject. Di Novi et al. (2019) observed that, although fiscal decentralization helps to contain disparities between regions, there is no statistical difference in the analyzed models. The results indicate that wealthier health regions tend to obtain better results with fiscal decentralization, indicating the reduction of inequities in health. Underprivileged regions, however, continue to rely on subsidy at central level, which does not render any effect for the RHA in terms of accountability and governance^[77]^.

Moreover, the study indicates that the significance of the impact of fiscal decentralization increases over the years^[77]^, supporting the idea that it takes time to observe the effects of regionalization on a population’s health. This finding corroborates the need for further studies on health regionalization, since the topic still has critical gaps that have not yet been exhausted by its scientific literature.

**Reviewer 1, comment 2:** “Accordingly, they should also acknowledge adherence to the research guidelines for systematic reviews, PRISMA, as in <http://www.prisma-statement.org/>.

**Our response:**

A new revision of the manuscript was carried out in order to comply with PRISMA for systematic review protocols.

**Reviewer 1, comment 3:** “One small comment regards the research question, for which I would recommend a slight rewording. "A systematic review was conducted to answer the following question: what are effective interventions to improve health and management indicators within the health services regionalization?" I recommend striking the word THE and instead it should read: "what are effective interventions to improve health and management indicators within health services regionalization?" I look forward to seeing the updates on this study after the revision of the search strategy.”

**Our response:**

We changed the text, as suggested.
